# Steerable microcatheter-assisted selective inflow embolization of a giant femoral artery bifurcation pseudoaneurysm: A case report

**DOI:** 10.1016/j.radcr.2026.07.018

**Published:** 2026-07-31

**Authors:** Fumie Sugihara, Misa Iwasaki, Sayaka Shirai, Tatsuo Ueda, Hidemasa Saito, Hiromitsu Hayashi, Shin-ichiro Kumita

**Affiliations:** Department of Radiology, Nippon Medical School, Tokyo, Japan

**Keywords:** Femoral artery pseudoaneurysm, Femoral artery bifurcation, Selective inflow embolization, *N*-Butyl cyanoacrylate, Steerable microcatheter

## Abstract

Femoral artery pseudoaneurysm is a known complication of arterial access, and its management can be particularly challenging in patients with severe coagulopathy or complex vascular anatomy. Here, we report a case of a giant femoral artery bifurcation pseudoaneurysm arising from a long arterial sheath puncture tract at the bifurcation of the superficial and deep femoral arteries in a critically ill patient with septic shock and coagulopathy. Selective catheterization of the narrow, irregular tract was achieved using a steerable microcatheter with coordinated 2-operator manipulation under real-time fluoroscopic guidance. Subsequent selective inflow embolization using a limited volume of a *N*-butyl cyanoacrylate–Lipiodol mixture successfully occluded the pseudoaneurysm without distal ischemic complications. This case demonstrates that advanced microcatheter techniques can enable safe and effective endovascular treatment of complex femoral artery pseudoaneurysms in high-risk patients.

## Introduction

Femoral artery pseudoaneurysm is a known complication of arterial access and catheter-based procedures [[1], [2], [3]]. Although most cases are successfully treated with ultrasound-guided compression or thrombin injection, these methods may be ineffective or contraindicated in patients with large pseudoaneurysms, severe coagulopathy, infection, or complex vascular anatomy [[1], [2], [3]].

Pseudoaneurysms arising from arterial sheath puncture tracts near bifurcation sites are particularly challenging to manage because of high-flow conditions and unfavorable vascular geometry and may occasionally be encountered in critically ill patients with limited treatment options [3]. In this context, endovascular treatment provides a minimally invasive alternative when conventional therapies are not feasible. Technological advances, including steerable microcatheters, may enhance the feasibility of selective catheterization in anatomically complex situations.

Here, we report a case of a giant femoral artery pseudoaneurysm at the bifurcation of the superficial and deep femoral arteries that was successfully treated with selective inflow embolization using a steerable microcatheter and coordinated two-operator manipulation.

## Case report

A 66-year-old man with recurrent pancreatic cancer presented with septic shock secondary to obstructive cholangitis, accompanied by coagulopathy resembling disseminated intravascular coagulation, which required daily platelet transfusions. A 4-Fr arterial sheath was placed in the right femoral artery for hemodynamic monitoring. After sheath removal, a groin hematoma developed rapidly. Doppler ultrasonography was initially performed and demonstrated a large groin hematoma with internal blood flow, confirming the diagnosis of a femoral artery pseudoaneurysm. During evaluation, the hematoma enlarged rapidly and the patient’s hemodynamic condition deteriorated. Because ongoing hemorrhage and possible retroperitoneal extension were suspected, contrast-enhanced computed tomography (CT) was performed for further assessment.

**Fig. 1:**
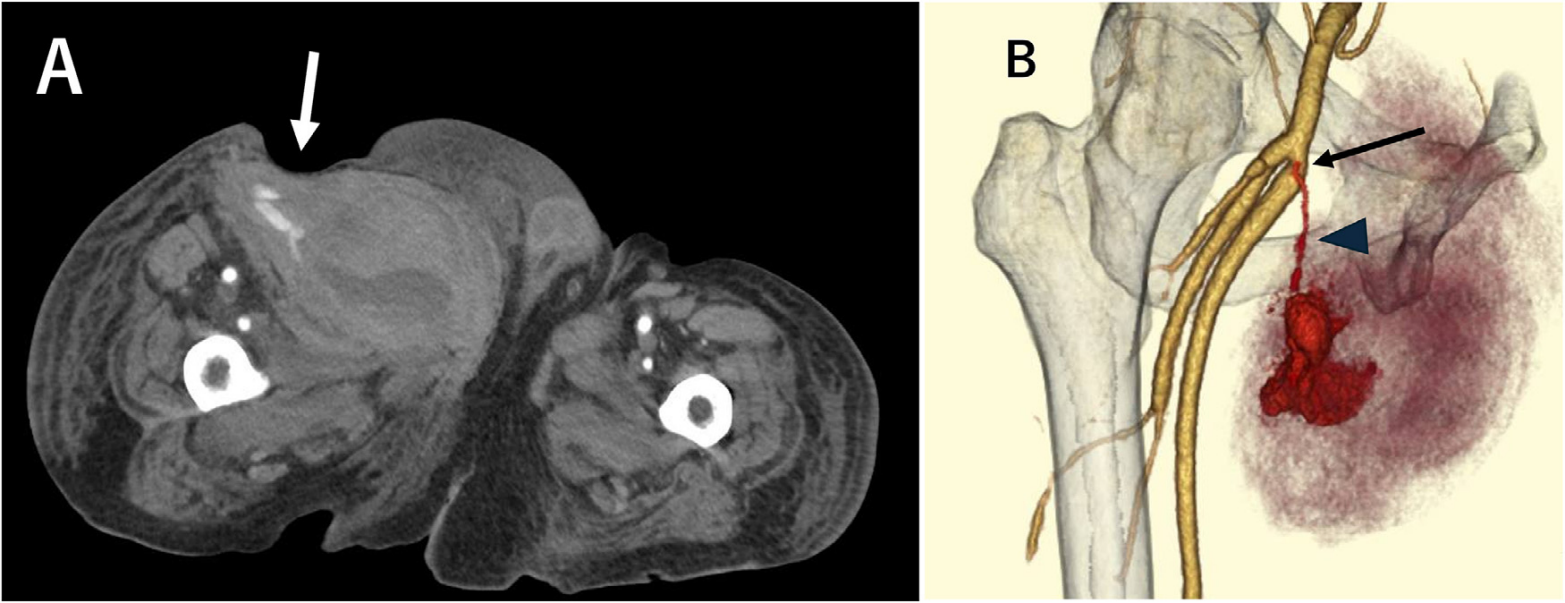
Initial imaging of a femoral artery pseudoaneurysm. (A) Contrast-enhanced computed tomography scan demonstrates a large femoral artery pseudoaneurysm with active contrast extravasation (arrow). (B) Three-dimensional, volume-rendered image of the pseudoaneurysm arising from a long arterial sheath puncture tract (arrowhead). The tract origin is indicated by an arrow near the bifurcation of the superficial and deep femoral arteries.

Contrast-enhanced CT revealed a large pseudoaneurysm (15 × 7 × 14 cm) arising from the femoral artery through a long puncture tract at the bifurcation of the superficial and deep femoral arteries (Fig. 1). Ultrasound-guided compression was unsuccessful. Ultrasound-guided thrombin injection was considered but deemed unsuitable due to poor sonographic visualization from the large pseudoaneurysm, which hindered reliable identification of the superficial and deep femoral arteries and raised concern for nontarget embolization. Surgical repair was contraindicated given the patient’s critical condition. Therefore, emergent endovascular treatment was performed.

**Fig. 2:**
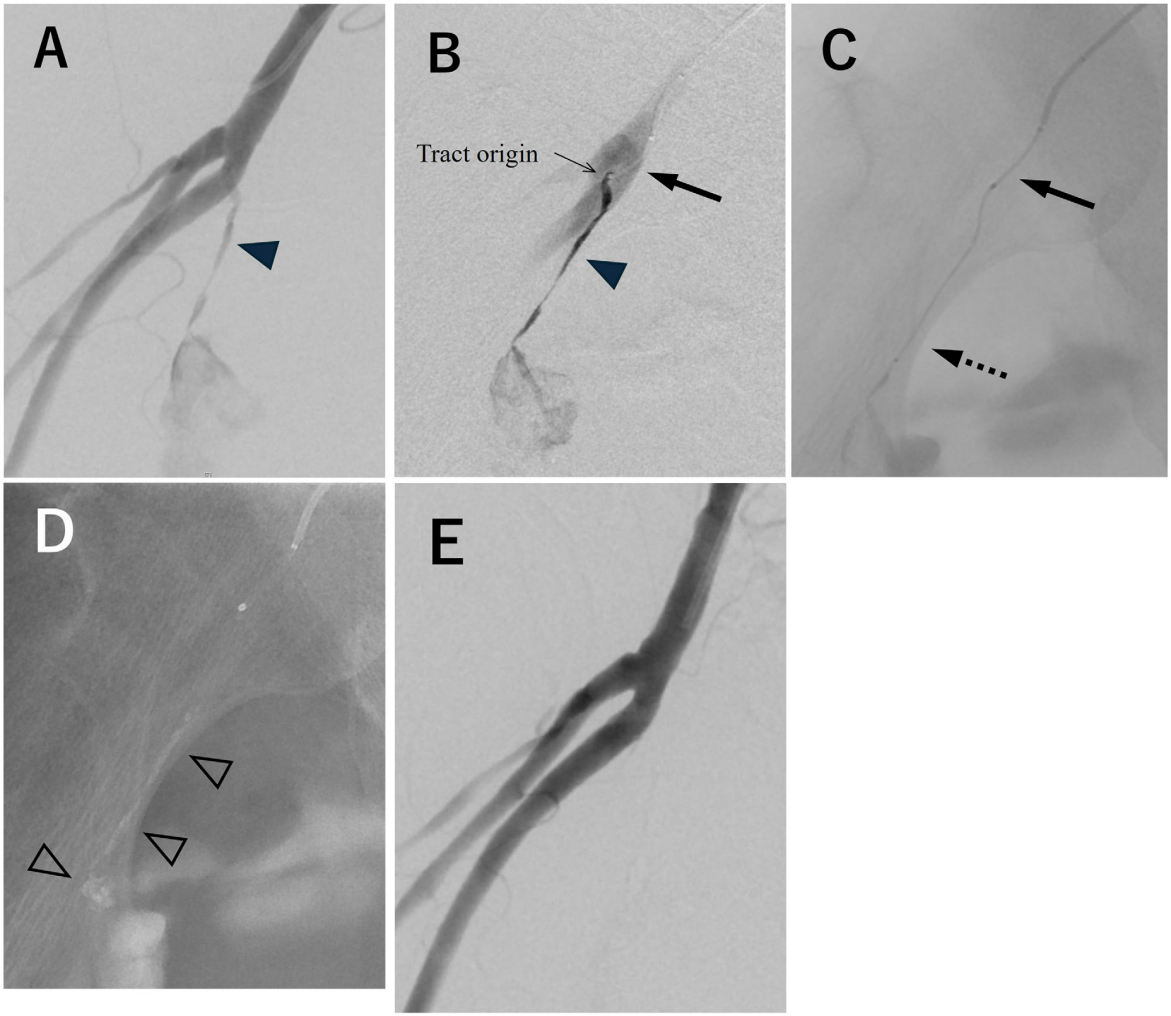
Selective inflow embolization using a steerable microcatheter. (A) Digital subtraction angiography demonstrates a pseudoaneurysm arising from a long arterial sheath puncture tract (arrowhead) at the bifurcation of the superficial and deep femoral arteries. (B) Selective angiography reveals an irregular arterial sheath puncture tract (arrowhead) with stenosis at its origin. Despite the unfavorable tract morphology, the tract origin was successfully cannulated using a 2.9-Fr steerable microcatheter (arrow). (C) Fluoroscopic image shows advancement of a 1.9-Fr microcatheter (dashed arrow) through the 2.9-Fr steerable microcatheter (arrow) toward the pseudoaneurysm entrance. (D) Postembolization fluoroscopy demonstrates the NBCA–Lipiodol cast (open arrowheads) within the embolized segment, extending from the pseudoaneurysm entrance into the sheath tract, with no extension to the tract origin. (E) Postembolization digital subtraction angiography confirms disappearance of the arterial sheath puncture tract and pseudoaneurysm.

A 5-Fr short sheath (Medikit, Miyazaki, Japan) was inserted via the left femoral artery, and a 4-Fr Cobra-type catheter (Medikit) was advanced to the right external iliac artery. Angiography demonstrated active extravasation through a narrow, irregular sheath tract approximately 1.4 mm in diameter and 4 cm in length (Fig. 2A). Selective catheterization was initially attempted using a 45° angled microcatheter (GOLD CREST Neo; Medicos Hirata, Osaka, Japan); however, the tract origin could not be selectively cannulated. A 2.9-Fr steerable microcatheter (Leonis Mova High Flow Type; Sumitomo Bakelite, Tokyo, Japan) was therefore used, allowing successful selective cannulation with coordinated two-operator manipulation under real-time fluoroscopic guidance (Fig. 2B).

An extension tube was connected to the hub of the steerable microcatheter so that the second operator could inject contrast medium via a 3-mL syringe without interfering with the primary operator’s two-handed catheter control. This arrangement enabled contrast injection and catheter manipulation to be performed simultaneously: the second operator repeatedly injected contrast medium to maintain real-time fluoroscopic visualization, while the primary operator instantly adjusted the catheter tip in response to the live fluoroscopic image. The steering dial was manipulated with the right hand, while the catheter shaft was advanced or withdrawn with the left hand, creating an endoscope-like two-handed control system for selective catheterization. When the tract origin was clearly visualized, the microcatheter tip was actively deflected using the steering dial to achieve cannulation of the tract origin, after which the steering dial stopper was locked to maintain directional stability. A 0.014-inch microguidewire (CHIKAI V; ASAHI INTECC, Aichi, Japan) was then gently advanced through the tract under fluoroscopic monitoring to avoid injury to the fragile tract. Subsequently, over the guidewire, a 1.9-Fr microcatheter (Carnelian Marvel; Tokai Medical Products, Aichi, Japan) was advanced through the steerable microcatheter and positioned near the pseudoaneurysm entrance (Fig. 2C). Magnified fluoroscopy was used during these steps; roadmap guidance was not employed. Systemic heparinization was withheld given the patient’s coagulopathy and active hemorrhage. Angiography demonstrated that the pseudoaneurysm was blind-ended, with no evidence of outflow or collateral drainage, suggesting that inflow occlusion alone would be sufficient. Approximately 1.2 mL of a 33% N-butyl cyanoacrylate (NBCA)–Lipiodol mixture was injected to embolize the pseudoaneurysm entrance and sheath tract while avoiding embolization of the tract origin (Fig. 2D). The embolization endpoint was complete disappearance of flow into the pseudoaneurysm through the sheath tract while preserving antegrade flow in both the superficial femoral artery and deep femoral artery. Distal embolic protection was not used because embolization was intentionally limited to the pseudoaneurysm entrance and sheath tract. The 33% NBCA–Lipiodol ratio (1:2) was empirically selected to balance adequate polymerization time for controlled embolization of the pseudoaneurysm entrance and adjacent tract with the need to minimize reflux or unintended proximal extension into the femoral artery. The total fluoroscopy time was 33 minutes, and the total iodinated contrast volume was approximately 200 mL.

Post-embolization angiography demonstrated complete disappearance of the pseudoaneurysm and the arterial sheath puncture tract (Fig. 2E).

**Fig. 3:**
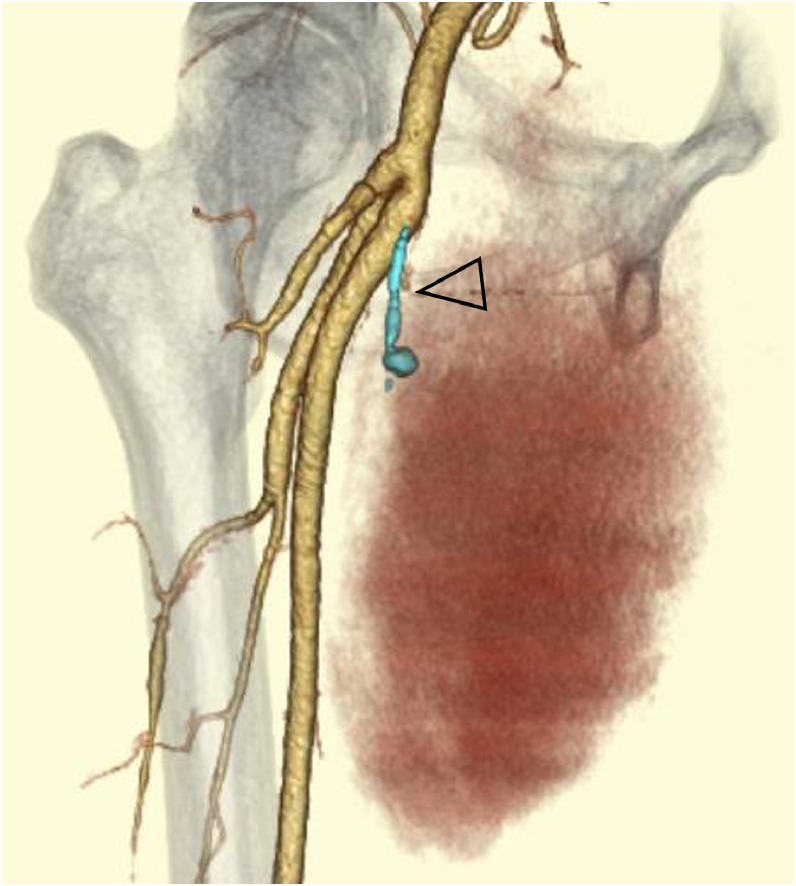
Durable embolization on follow-up computed tomography (CT) scan. Follow-up contrast-enhanced CT shows complete disappearance of the extravasation and tract origin, with persistent NBCA–Lipiodol cast (open arrowhead) along the embolized tract.

Follow-up contrast-enhanced CT performed 9 days later demonstrated resolution of extravasation and durable occlusion of the tract with preserved distal perfusion (Fig. 3). During the subsequent clinical course, no recurrent hemorrhage, clinical signs of limb ischemia, or evidence of pseudoaneurysm recurrence were observed. The patient died 7 weeks after the procedure because of rupture of an infectious cerebral aneurysm unrelated to the present intervention.

## Discussion

Femoral artery pseudoaneurysm is a well-recognized complication following arterial puncture, and endovascular treatment is considered when standard nonsurgical therapies are contraindicated or unsuccessful [4,5]. In the present case, severe septic shock and coagulopathy precluded surgical repair, while the large pseudoaneurysm with poor sonographic visualization made ultrasound-guided compression and thrombin injection unsuitable [6], leaving catheter-based intervention as the only feasible option.

Femoral pseudoaneurysms are commonly managed with ultrasound-guided thrombin injection, which is currently regarded as the first-line treatment owing to its high technical success rate [[5], [6], [7]]. Surgical repair and covered stent placement remain important alternatives when thrombin injection is contraindicated or unsuccessful [5,8]. Therefore, treatment selection should be individualized according to pseudoaneurysm morphology, vascular anatomy, and patient condition [5].

The major technical challenge was the complex anatomy at the bifurcation of the superficial and deep femoral arteries under high-flow conditions [3]. In addition, the sheath tract was narrow and morphologically irregular, particularly at its origin, resulting in catheter instability and difficulty in selective cannulation. Balloon-assisted embolization has been reported as an effective flow-control technique [1,2]. However, in a Y-shaped bifurcation, a single balloon may not reliably achieve complete flow occlusion and may therefore be insufficient. Moreover, because balloon assistance would have required insertion of an additional sheath or exchange to a larger-caliber sheath, potentially increasing the risk of access-related complications, it was reserved as a last-line option.

In this case, stent-graft placement was also considered as a potential therapeutic option [4,5,8]. However, because the pseudoaneurysm originated at the femoral artery bifurcation, effective occlusion would require deployment of a stent-graft extending from the common femoral artery to the superficial femoral artery, thereby sacrificing the deep femoral artery. Furthermore, the femoral artery lies at a flexion point and is subject to significant mechanical stress, raising concerns about long-term durability, including stent fracture and in-stent restenosis [4,5,8]. Based on these anatomical and biomechanical considerations, stent-graft placement was not considered an appropriate first-line treatment in this case. Similarly, a recent study emphasized the importance of individualized endovascular treatment strategies in complex vascular pathologies when conventional surgical or endovascular options are limited by anatomical or clinical considerations [9].

In this context, the use of a steerable microcatheter facilitated selective catheterization [10,11]. In the present case, coordinated 2-operator manipulation was employed to enable simultaneous catheter manipulation and contrast injection. By connecting an extension tube to the catheter hub, the second operator could repeatedly inject contrast medium without interfering with the primary operator’s 2-handed catheter control. This arrangement allowed the primary operator to manipulate the steerable microcatheter using an endoscope-like 2-handed control system while maintaining continuous real-time fluoroscopic visualization. In conventional single-operator technique, the operator must temporarily interrupt catheter control to inject contrast medium, making simultaneous visualization and precise catheter manipulation difficult. In the present case, continuous real-time visualization and uninterrupted catheter control were particularly important because the tract origin was narrow and morphologically irregular, requiring precise real-time adjustments of catheter position and orientation for successful selective catheterization.

Although dual-operator techniques have been described for dual-lumen microcatheters [12], a coordinated two-operator strategy using a steerable microcatheter has not been widely described. This approach may be particularly useful in cases involving narrow, tortuous, or morphologically complex target vessels where continuous visualization and uninterrupted catheter control are essential, whereas conventional single-operator manipulation may be sufficient in less challenging anatomy. However, given that this report describes a single experience, broader clinical validation is required before generalizable conclusions can be drawn regarding the safety and reproducibility of the coordinated two-operator technique using a steerable microcatheter. This technique also requires additional personnel and familiarity with steerable microcatheter manipulation. Furthermore, successful implementation depends on close coordination between operators, and repeated contrast injections to maintain real-time fluoroscopic visualization may increase overall contrast utilization compared with conventional single-operator manipulation.

Regarding the embolization strategy, selective inflow embolization targeting the pseudoaneurysm entrance and sheath tract was performed rather than complete filling of the aneurysm sac [13]. Angiography demonstrated a blind-ended pseudoaneurysm without evidence of outflow or collateral drainage, suggesting that inflow occlusion alone would be sufficient to achieve thrombosis. During NBCA injection, care was taken to avoid embolization at the tract origin to reduce the risk of nontarget embolization [[1], [2], [3]]. Distal embolic protection was not used because embolization was intentionally limited to the pseudoaneurysm entrance and sheath tract through selective microcatheter positioning. Furthermore, distal protection would have required placement of an additional sheath or larger access system, potentially increasing the risk of access-related complications [1]. Therefore, the anticipated benefits of distal protection were not considered to outweigh the additional procedural complexity or the potential access-related risks. Accordingly, complete occlusion of the pseudoaneurysm was achieved without distal ischemic complications.

This case suggests that selective inflow embolization using a steerable microcatheter may be a useful option for the treatment of complex femoral artery pseudoaneurysms in high-risk patients when conventional therapies are not feasible.

## Conclusion

Selective inflow embolization using a steerable microcatheter with coordinated two-operator manipulation enabled successful treatment of a complex femoral artery pseudoaneurysm in a critically ill patient. This approach may represent a useful salvage option when conventional therapies are not feasible and highlights the evolving role of advanced catheter technologies in endovascular practice. Further clinical experience is required to assess reproducibility and long-term outcomes.

## Declaration of generative AI and AI-assisted technologies in the manuscript preparation process

During the preparation of this manuscript, the authors used a large language model (ChatGPT, OpenAI) in order to gain assistance with language editing and improvement of manuscript clarity. After using this tool, the authors reviewed and edited the content as needed and take full responsibility for the content of the published article.

## Patient consent

Informed consent was obtained from the patient for publication of the report and associated images.
